# Healthy food and determinants of food choice on online food delivery applications

**DOI:** 10.1371/journal.pone.0293004

**Published:** 2023-10-19

**Authors:** Tareq M. Osaili, Anas A. Al-Nabulsi, Asma’ O. Taybeh, Leila Cheikh Ismail, Sheima T. Saleh

**Affiliations:** 1 Department of Nutrition and Food Technology, Faculty of Agriculture, Jordan University of Science and Technology, Irbid, Jordan; 2 Department of Clinical Nutrition and Dietetics, College of Health Sciences, University of Sharjah, Sharjah, United Arab Emirates; Gazi University, Faculty of Health Sciences, TURKEY

## Abstract

Online food delivery applications (OFD apps) provide consumers with a wide range of options to choose from. The present study aimed to assess the usage of OFD apps and investigate the factors that affect food choices with a special emphasis on healthy food choices and hygiene. A cross-sectional study among food delivery application users in Jordan was conducted using an online questionnaire between March and May 2022. A total of 675 eligible subjects participated in the study. Consumers’ demographic characteristics, data on consumers’ use of OFD apps, consumers’ perceptions of healthy food availability in OFD apps, and consumers’ attitudes toward food safety and delivery hygiene were collected and analyzed. About 64% of the studied sample used OFD apps weekly. Fast food was the most popular option for ordering (87.1%) and lunchtime was the most preferred time to order food (67.3%) for most of the respondents. Respondents’ perceptions of a “healthy meal” was associated with the presence of a variety of vegetables in the meal. Food price, food appearance, time of delivery, macronutrient content information, the availability of healthy options, and considering vegetables as part of a healthy meal were determinants of consumer food choice (p<0.05). The findings suggest that the online food environment in Jordan was perceived to be unhealthy. Nevertheless, the convenient nature and the popularity of OFD apps hold great potential to promote healthy eating among consumers.

## Introduction

The usage of online services through mobile applications has increased tremendously over the past decade [1]. Given this increase in usage, some features of the food environment have been digitalized to improve food production and distribution. Digital food environment is defined as the online setting that provides a variety of services and information, through which consumers’ food and nutrition choices and behaviors are influenced and directed. The digital food environment includes social media, digital food marketing, online food retail and digital health promotion interventions [2]. The physical and the digital food environments are interconnected and are influencing one another, for instance, the digitalized food environment enabled the emergence of new styles of selling and buying food, and eventually changing availability of food, affecting the physical distance to shops and the time allocated for shopping [2].

Estimates show that the top ten downloaded applications by Google Play and Apple App store users were related to food delivery [3, 4]. Online food delivery applications (OFD apps) are innovative and convenient channels where consumers can easily access restaurant menus and get the food ordered at their desired location [5]. The popularity of OFD apps could be attributed to the quick and easy method of order placement, nonexistent queues, and the option of contactless payment [6, 7]. OFD apps are designed to meet the needs of a specific restaurant or a food joint and such applications are usually under the control of the food organization. However, especially due to travel constraints related to the COVID-19 pandemic, third-party food delivery services with multiple restaurant partnerships have grown exponentially, with a primary responsibility of managing the logistics of food delivery [8]. The nature of this business partnership is usually more profitable for restaurants when compared to individual company-associated food home delivery and provides more leverage to consumers [9].

The COVID-19 pandemic significantly affected consumers’ relationship with food and eating [10]. During the pandemic, and due to the strict worldwide lockdown measures and mobility restrictions, the hospitality industry was strongly affected [11]. OFD apps have become a prominent source of ordering food as people cannot dine in restaurants and are restricted from using public services [12]. And so, some behavioral changes resulting from the pandemic may continue even after it ends [13].

Busy lifestyles have been associated with alterations in consumer eating practices and dietary habits [14]. Consumers nowadays are more health conscious and aware of healthy eating due to the global increase in nutrition-related disorders (e.g. obesity, hypertension, diabetes, etc) [15]. Non-communicable diseases (NCDs) are the leading cause of morbidity and mortality worldwide. In Jordan in 2016, it was estimated that 78% of the total deaths (36,000) were NCDs, mainly cardiovascular diseases (37%), cancer (12%), and diabetes (6%) [16]. High triglyceride (56.5%), cholesterol (39.5%), hypertension (28.6%), and diabetes (22.3%) are the leading causes of morbidity among Jordanians aged 25 years and above, with the associated risk factors being physical inactivity, unhealthy diet and smoking [16]. A healthy diet pattern would incorporate fruits, vegetables, and proteins, and would limit undesirable food items like saturated and trans fats and sodium [17]. The success of the sales of healthy food items delivered through third-party delivery services is primarily dependent on two factors which are consumer willingness and the variety in the availability of healthy foods. Consumers willing to eat healthy foods usually face a paradox of insufficient healthy food options available in food delivery applications [15]. On the contrary, OFD apps may felicitate their consumption of unhealthy foods and thus adversely impact their health status [18].

The healthiness of food is not limited to the variety of the nutrients it provides but extends also food safety aspects including proper food preparation and handling [19]. Healthy food prepared in an unhygienic manner may adversely impact the health of the consumer and may lead to foodborne disease outbreaks. Information about employee hygiene, food handling and cleanliness of the establishment is not usually provided to consumers when they order from OFD apps [20]. Therefore, it is highly unlikely that consumers who order food online would be aware of the hygienic status of the restaurant or the food being prepared.

Jordan is a country with a population of 10 million located in the Levant region. Online food delivery services are a relatively new concept in the country compared to other neighboring regional territories such as Kuwait, Saudi Arabia, Qatar, and the United Arab Emirates. However, by the end of 2018, more than 700 restaurants had joined Talabat; an online food ordering company and commenced serving consumers in Amman, the capital city of Jordan [5]. Moreover, to reach a much larger audience, many restaurants developed online food ordering applications in dual languages (Arabic and English) [5]. To date, most of the OFD apps in Jordan such as Talabat, Uber Eats, and Careem Now are mainly operating in selected cities namely, Amman, Irbid, Aqaba (the coastal and a leading tourist city), Zarqa, Salt, and Madaba.

To the best of our knowledge, there is no available literature on OFD apps usage in Jordan. Thus, this study aimed to assess the usage of OFD apps and investigate the factors that affect the usage of these platforms with a special emphasis on healthy food choices and hygiene.

## Methods

### 1. Study design and sample collection

A population, web-based cross-sectional study was conducted in Jordan between March and May 2022. Inclusion criteria were adults who are ≥18 years, currently residing in Jordan, and who use OFD apps at least once a month. Food delivery drivers and those who refused to participate were excluded from the study. Convenience sampling, facilitated by the snowball sampling technique, was employed to recruit participants. This method was used to allow for quicker access to participants, particularly when with resource constraints, logistical challenges, or time limitations. Participants were recruited by sharing a web link connecting to the online survey via e-mail invitations with the contact lists being gathered from personal and professional contacts of the research team, including colleagues, friends, and family members. In addition to the email invitations, the survey link was shared and posted through the authors own personal and professional social media accounts; e.g., LinkedIn™, Facebook™, and WhatsApp™. The authors also requested the initial participants to share the survey link with their own networks to help recruit future subjects, who, in turn, forwarded the link to their own contacts. This approach facilitated the inclusion of those who might have not been directly reached through the research team but were interested in participating in the study. The first page of the online survey included the information sheet which provided a short and clear description of the study and its objectives. Also, it included screening questions to ensure that the participants aged ≥18 years old, currently reside or live in Jordan, and are not OFD apps drivers. The participants were informed that their participation was voluntary and that they could withdraw at any point. No identifiable personal information was collected and participants were required to check the box “Agree” to confirm reading the information sheets and to proceed with the survey questions. The survey completion time ranged from 3–5 minutes and participants were required to answer all questions so that no data were missing.

### 2. Questionnaire design and validation

The data collection tool was developed by the researchers upon review of the available relevant literature [21–23]. The first draft of the questionnaire was prepared in English and included 35 close-ended questions (Likert-scale, Dichotomous questions, Multiple choice questions, Checklist type multiple choice questions).

For the validation of the questionnaire, the first version of the questionnaire was evaluated by a panel of four academicians. The academicians have a professional background in nutrition and food safety with a long track record in survey research in the field of food safety, nutrition, and public health. The reviewers were asked to rate the questions on a Likert scale of 1 to 10 for language, clarity, repetition, and content and determine if the questions met the objectives of the study. Any question with an average score of less than 70% was amended/removed. Any other suggestions from the reviewers were considered. The questionnaire reliability was evaluated using Cronbach’s alpha coefficient for internal consistency generating a Cronbach’s alpha value of (0.751) indicating an acceptable internal consistency. The questionnaire was then translated into Arabic and back-translated to English by three bilingual experts to determine the accuracy of the translation. The final version of the questionnaire was composed of 27 questions divided into four sections. The first section inquired about the demographic characteristics of the participants (7 questions). The second section investigated the use of OFD apps (5 questions). The third section explored participants’ perceptions of healthy choices availability on OFD apps (8 questions). The last section was comprised of 7 questions related to food safety and delivery hygiene. The final version of the questionnaire was prepared on Google Forms; a survey administration software included in the Google Drive office suite. The survey was pilot tested with 10 people and no further amendments were implied.

### 3. Ethical approval

The ethical approval for the research was granted by the Institutional Review Board (IRB) at Jordan University of Science and Technology Human Ethical Committee (Ref#: 12/150/2022). An electronic consent form was obtained from all participants.

### 4. Statistical analysis

IBM Statistical Package for Social Sciences (SPSS, version 25.0) was used to analyze the collected data. Descriptive statistics of means, standard deviation, frequencies, and percentages were used for variables as appropriate. Associations between categorical variables and healthy food choices were explored using a chi-square test. The attitudes towards food safety and delivery hygiene were measured using a 3-point Likert Scale (1 =  disagree, 2 = neutral, 3 = agree) and were considered as categorical. Chi-square test was conducted to examine the association between the participants’ demographic characteristics and their attitudes towards food safety and delivery hygiene. Binary logistic regression was used to understand whether looking for healthy food choices can be predicted based on different variables. In examining the predictive factors on the choice to seek healthy food options, binary logistic regression was employed. The dependent variable was the inclination to “look for healthy food choices,” assessed dichotomously as “yes” or “no.” Independent variables were food appearance, price, time of delivery, healthy options availability, macronutrient content labeling and variety of vegetables content. To evaluate the model’s performance, odds ratio, confidence intervals and p-values were measured. A p-value of less than 0.05 was considered to be statistically significant.

## Results

### 1. Demographic characteristics

Of the 834 prospective respondents who followed the link, only those who responded positively to using OFD apps were selected for the survey, resulting in a final sample size of 675 for empirical analysis. As presented in Table 1, about two-thirds of the participants were females (69.9%), and aged 18–25 years old (65.6%). The majority had a bachelor’s degree (78.2%) and lived in the central region of Jordan (71.3%) and had a monthly allowance of 300 Jordanian dinars ($423.14) or less (59.0%). Concerning OFD apps, more than a third of the participants (36.4%) used OFD apps once a month while 27.9% used them once a week. The most popular OFD app among the participants was Talabat (92.9%) followed by Careem NOW (27.4%), whereas other applications were far less popular (9.1%).

**Table 1:** Demographic characteristics of the study participants (n = 675).

Character		n	%
**Gender**			
	Female	472	69.9
	Male	203	30.1
**Age**			
	18–25	443	65.6
	26–35	145	21.5
	36–45	44	6.5
	46–55	24	3.6
	>55	19	2.8
**Educational level**			
	High school or less	46	6.8
	Diploma	40	5.9
	Bachelor’s degree	528	78.2
	Postgraduate	61	9.0
**Residency**			
	North Region	138	20.4
	Central Region	481	71.3
	South Region	56	8.3
**Monthly allowance/income**			
	<100 JD ^a^	191	28.3
	100–300 JD	207	30.7
	301–500 JD	121	17.9
	501–1000 JD	96	14.2
	>1000 JD	60	8.9
**OFD apps usage frequency**			
	Once a month	246	36.4
	Once a week	188	27.9
	2–3 times/ week	172	25.5
	4–6 times/ week	46	6.8
	Daily	23	3.4
**Applications brands** *			
	Talabat	627	92.9
	Uber Eats	15	2.2
	Careem NOW	185	27.4
	Eat delivery	22	3.3
	Other	24	3.6

*Multiple responses were allowed; the total number of responses is greater than the number of surveyed participants

ª Jordanian Dinar (JD)

### 2. OFD apps usage

Participants were asked about the different types of cuisine they look for when ordering meals online (Table 2). Fast food was the most in-demand (87.1%), followed by desserts (39.3%), Arab cuisine (22.4%), and Italian cuisine (19.9%). Keto and vegetarian foods were the least ordered types of food to order (13.1% and 5.6%, respectively). Participants were most likely to order food for lunch (67.3%) and dinner (60.6%) while a far lesser proportion used OFD apps to order breakfast (9.3%).

**Table 2:** Factors affecting the usage of OFD apps (n = 675).

Item		n	%
What cuisine do you mostly look for when using OFD apps? *			
	Vegetarian/vegan	38	5.6
	Fast food	588	87.1
	Asian cuisine	103	15.3
	Italian cuisine	134	19.9
	Local cuisine	94	13.9
	Arabian cuisine	151	22.4
	Salads	83	12.3
	Keto food	21	3.1
	Desserts	265	39.3
**Which of the following meals/items do you order mostly using OFD apps?** *			
	Breakfast	63	9.3
	Lunch	454	67.3
	Dinner	409	60.6
	Snacks	103	15.3
	Late night snacks	165	24.4
	Drinks	63	9.3
Factors affecting your food choice when using OFD apps*			
	Food appearance	385	57.0
	Price	479	71.0
	Time of delivery	344	51.0
	Healthy options availability	122	18.1
	Hygienic status of the restaurants	381	56.4
	Display Calorie content (Kcal)	249	36.9
	Display Macronutrient content (protein, fat, carbohydrates)	274	40.6
**I have been influenced by social media posts, magazines, TV, or YouTube advertisements to use OFD apps**			
	Disagree	81	12.0
	Neutral	179	26.5
	Agree	415	61.5
**Friends and family have influenced my choice of OFD apps**			
	Disagree	101	15.0
	Neutral	147	21.8
	Agree	427	63.3

*Multiple responses were allowed; the total number of responses is greater than the number of surveyed participants

Regarding food choice determining factors, price was the most reported influencing food choice factor (71.0%), followed by food appearance (57.0%), the hygienic status of the restaurant (56.4%), and delivery time (51.0%). Moreover, 40.6% and 36.9% of the participants were influenced by the information about nutrient and calorie content, respectively. Only 18.1% of the participants reported being influenced by the availability of healthy options. Further, more than sixty percent of the participants agreed that they have been influenced by social media posts, magazines, TV, or YouTube advertisements to use food delivery applications (61.5%) and a similar number (63.3%) of participants agreed that they have been influenced by their friends and family (Table 2).

### 3. Consumer perception on placing healthy food orders

Almost one in four of the participants reported looking for healthy food choices when ordering through OFD apps. Table 3 shows the participants’ agreement on statements about ordering healthy food through the applications. About two-thirds of the participants agreed that it was difficult to find healthy choices in OFD apps (63.4%). Moreover, more than half of the participants agreed that OFD apps had increased their food intake and appetite (57.6%) and a similar proportion reported that their eating habits have changed due to OFD apps (57.8%). About a third of the participants reported not ordering a healthy side dish when using OFD apps, however, a similar proportion agreed that OFD apps had made them aware of healthier alternatives that they did not consider previously. Furthermore, only a third of the participants favored paying a higher price for healthy food options.

**Table 3:** Consumer perception on ordering healthy food (n = 675).

	N (%)		
Item	Agree	Neutral	Disagree
I often find it difficult to find healthy food choices on OFD apps	428 (63.4)	202 (29.9)	45 (6.7)
I feel that ordering from OFD apps has increased my food intake and appetite	389 (57.6)	164 (24.3)	122 (18.1)
Using OFD apps has changed my eating habits (for example: having late-night meals, or eating alone)	390 (57.8)	126 (18.7)	159 (23.6)
When using OFD apps, I usually order a healthy side dish (for example salads or vegetable soup)	239 (35.4)	196 (29.0)	240 (35.6)
Using OFD apps made me aware of healthier food alternatives that I did not consider before	247 (36.6)	213 (31.6)	215 (31.9)
I am willing to pay a higher price to get a healthier food choice	220 (32.6)	219 (32.4)	236 (35.0)

**Fig 1:**
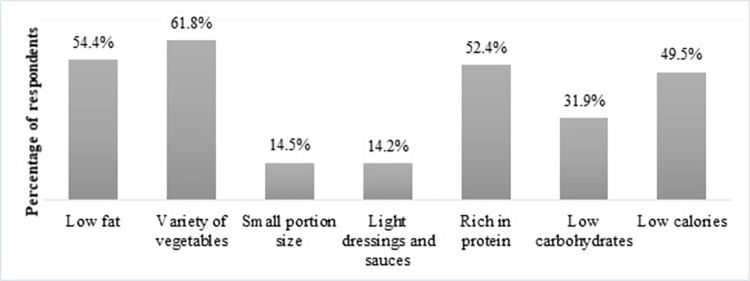
Perceptions of a healthy meal among study participants (n = 675).

When asked about what constitutes a healthy meal, participants’ perception comprised a meal abundant in vegetables (61.8%) and protein (52.4%) but low in fat (54.4%) as shown in Fig 1. Nearly half (49.5%) of the participants considered a lower calorie content as part of a healthy meal. About 14% of the participants believed that small-portion size meals and meals with light dressings and sauces were healthy.

The association between whether participants look for healthy food options or not and different study variables are shown in Table 4. A chi-square analysis revealed significant associations between; age (p = 0.017), food appearance (p = 0.030), price (p<0.001), time of delivery (p = 0.001), healthy options availability (p<0.001), availability of calories and macronutrient information (p = 0.019 and p = 0.001, respectively), and considering healthy food choices upon placing a food order. Significant factors were subjected to the binary logistic regression analysis (Table 5) to understand whether looking for healthy food choices can be predicted based on different variables (the dependent variable is "looking for healthy food choices", measured on a dichotomous scale–"yes" or "no"–and the independent variables: "factors influencing food choices" and "perception of a healthy meal). Logistic regression results indicated that participants who are not influenced by food appearance (OR: 1.494, 95% CI: 1.016–2.195), price (OR: 2.079, 95% CI: 1.386–3.119) and time of delivery (OR: 1.734, 95% CI: 1.177–2.556) were significantly associated with higher odds of looking for a healthy food option. Moreover, those who are not influenced by the availability of healthy food options were 0.2 times less likely to look for a healthy food option (OR: 0.209, 95% CI: 0.133–0.329). Also, the likelihood of looking for healthy food was lower among participants who are not influenced by the availability of macronutrient information than those who took into account the macronutrient content (OR: 0.593, 95% CI: 0.360–0.975). On the other hand, participants who do not consider the variety of fresh vegetables in a meal to be healthy are 1.5 times more likely to look for healthy food choices (OR: 1.496, 95% CI: 1.045–2.123).

**Table 4:** Factors influencing the choice of ordering healthy meals from OFD apps (n = 675).

		Looking for healthy choices when ordering		P-value
		Yes (n = 174) (%)	No (n = 501) (%)	
**Consumers characters**				
Gender	Female	115 (66.1)	357 (71.3)	P = 0.201
	Male	59 (33.9)	144 (28.7)	
Age	18–25	108 (62.1)	335 (66.9)	P = 0.017*
	26–35	32 (18.4)	113 (22.6)	
	36–45	14 (8.0)	30 (6.0)	
	46–55	11 (6.3)	13 (2.6)	
	>55	9 (5.2)	10 (1.9)	
Educational level	High school or less	13 (7.5)	33 (6.6)	P = 0.684
	Diploma	13 (7.5)	27 (5.3)	
	Bachelor’s degree	131 (75.3)	397 (79.2)	
	Postgraduate	17 (9.7)	44 (8.9)	
Residency	North Region	39 (22.4)	99 (19.8)	P = 0.214
	Central Region	116 (66.7)	365 (72.8)	
	South Region	19 (10.9)	37 (7.4)	
Monthly allowance	<100 JD	46 (26.4)	145 (28.9)	P = 0.408
	100–300 JD	48 (27.6)	159 (31.7)	
	301–500 JD	32 (18.4)	89 (17.8)	
	501–1000 JD	27 (15.5)	69 (13.8)	
	>1000 JD	21 (12.1)	39 (7.8)	
Using frequency	Once a month	62 (35.6)	184 (36.7)	P = 0.873
	Once a week	47 (27.0)	141 (28.1)	
	2–3 times/ week	44 (25.3)	128 (25.6)	
	4–6 times/ week	15 (8.6)	31 (6.2)	
	Daily	6 (3.5)	17 (3.4)	
**Factors influencing food choices**				
Food appearance (yes, n = 385)		87 (22.6)	298 (77.4)	P = 0.030*
Price (yes, n = 479)		100 (20.9)	379 (79.1)	P = 0.000*
Time of delivery (yes, n = 344)		70 (20.3)	274 (79.7)	P = 0.001*
Healthy options availability (yes, n = 122)		65 (53.3)	57 (46.7)	P = 0.000*
Hygienic status of the restaurants (yes, n = 381)		90 (23.6)	291 (76.4)	P = 0.145
Calorie content information (yes, n = 249)		77 (30.9)	172 (69.1)	P = 0.019*
Macronutrient content information (yes, n = 274)		89 (32.5)	185 (67.5)	P = 0.001*
**Perception of a healthy meal**				
Low fat (yes, n = 367)		96 (26.1)	271 (73.9)	P = 0.805
Variety of vegetables (yes, n = 417)		95 (22.8)	322 (77.2)	P = 0.024*
Small portion size (yes, n = 98)		28 (28.6)	70 (71.4)	P = 0.494
Light dressings and sauces (yes, n = 96)		31 (32.3)	65 (67.7)	P = 0.115
Rich in protein (yes, n = 354)		89 (25.1)	265 (74.9)	P = 0.691
Low carbohydrates (yes, n = 215)		58 (27.0)	157 (73.0)	P = 0.626
Low calories (yes, n = 334)		79 (23.7)	255 (76.3)	P = 0.212

* *p* value was based on the chi-square test at a 5% level.

**Table 5:** Regression analysis of the potential food and perception-related factors influencing participants’ food choices.

	**OR (CI)**	**P-value**
**Factors influencing food choices**		
Food appearance	1.494 (1.016–2.195)	P = 0.041*
Price	2.079 (1.386–3.119)	P = 0.000*
Time of delivery	1.734 (1.177–2.556)	P = 0.005*
Healthy options availability	0.209 (0.133–0.329)	P = 0.000*
Macronutrient content (protein, fat, carbohydrates)	0.593 (0.360–0.975)	P = 0.039*
	**OR (CI)**	**P-value**
**Perception of a healthy meal**		
Variety of vegetables	1.496 (1.045–2.123)	P = 0.024*

* *p* value was based on binary logistic regression test at 5% level.

OR: Odds Ratio, CI: Confidence Interval. Dependent variable: looking for healthy food choices, Independent variables: factors influencing food choices and perception of a healthy meal.

### 4. Food safety and delivery hygiene

Participants were requested to rate their agreement on seven statements about food safety and delivery hygiene in OFD apps using a 3-point Likert Scale (1 =  disagree, 2 = neutral, 3 = agree) as shown in Table 6. The results revealed that the majority (88.9%) agreed that the hygiene rating factor feature for restaurants in OFD apps is useful while ordering. Further analysis (Chi-square test) revealed a significant association (p = 0.040) between participants’ residency place and their agreement that having the hygiene rating factor of the restaurant in the food application would be useful when ordering as shown in Table 7. Only around half (51.6%) of the surveyed subjects believed that the food is prepared and delivered under sanitary conditions and the chi-square test results showed a significant association between participants’ gender (p = 0.049) and their agreement to this statement. The majority of the surveyed participants believed that the temperature of the meal upon delivery gives an impression about the quality (89.6%) and safety (80.7%) of the meal. Further analysis revealed a significant association between agreement to these statements and participants’ age (p = 0.017, p = 0.00), respectively. More than three-quarters (77.6%) agreed that the packaging of the meal influenced their food choices, and more than sixty percent (63.7%) showed a positive attitude towards environmentally friendly packages. A significant association between participants’ frequency of using OFD apps and their attitudes of believing that the use of environmentally friendly packaging materials influences their food choices (p = 0.033) was observed. Moreover, results unveiled statistically significant association (p = 0.038) between participants’ place of residence and the influence of meal packaging on their food choices.

**Table 6:** Participants attitudes towards food safety and delivery hygiene while ordering food through OFD apps (n = 675).

	Agree	Neutral	Disagree
Item	n (%)		
I believe that having the hygiene rating factor of the restaurant in the food application would be useful when ordering	600 (88.9)	61 (9.0)	14 (2.1)
I believe that the items available are prepared and delivered under sanitary conditions	348 (51.6)	274 (40.6)	53 (7.9)
I believe that the packaging of the meal influences my food choice	524 (77.6)	106 (15.7)	45 (6.7)
I believe that having the meal delivered in environmentally friendly packaging materials influences my food choice	430 (63.7)	167 (24.7)	78 (11.6)
The appearance of the driver (cleanliness, neatness) affects my perception of the meal’s hygiene	483 (71.6)	122 (18.1)	70 (10.4)
The temperature of the meal when delivered mainly gives me an impression about the quality of the food	605 (89.6)	35 (5.2)	35 (5.2)
The temperature of the meal when delivered mainly gives me an impression about the safety of the meal	545 (80.7)	79 (11.7)	51 (7.6)

**Table 7:** Association between participant’s socio-demographic characteristics and their attitudes towards food safety and delivery hygiene while ordering through OFD apps.

Item	Gender	Age	Educational level	Residency	Monthly allowance/income	OFD apps usage frequency
I believe that having the hygiene rating factor of the restaurant in the food application would be useful when ordering	P = 0.080	P = 0.354	P = 0.861	P = 0.040*	P = 0.469	P = 0.558
I believe that the items available are prepared and delivered under sanitary conditions	P = 0.049*	P = 0.260	P = 0.614	P = 0.817	P = 0.911	P = 0.152
I believe that the packaging of the meal influences my food choice	P = 0.175	P = 0.158	P = 0.635	P = 0.038*	P = 0.661	P = 0.501
I believe that having the meal delivered in environmentally friendly packaging materials influences my food choice	P = 0.865	P = 0.438	P = 0.250	P = 0.098	P = 0.486	P = 0.033*
The appearance of the driver (cleanliness, neatness) affects my perception of the meal’s hygiene	P = 0.402	P = 0.249	P = 0.204	P = 0.163	P = 0.572	P = 0.105
The temperature of the meal when delivered mainly gives me an impression about the quality of the food	P = 0.614	P = 0.017*	P = 0.508	P = 0.140	P = 0.062	P = 0.293
The temperature of the meal when delivered mainly gives me an impression about the safety of the meal	P = 0.135	P = 0.000*	P = 0.475	P = 0.066	P = 0.653	P = 0.141

*Significance level at P < 0.05.

## Discussion

The present study highlighted the perceptions of OFD apps among consumers in Jordan. It also examined factors of OFD apps utilization with a secondary focus on healthy food options and food safety/delivery hygiene.

OFD apps represent an important dimension of the digital food environment. Amongst the different OFD apps referred to in this study, Talabat was the most popular choice among the participants. Talabat is the largest online food delivery platform in the Middle East, which was founded in Kuwait in the year 2004 [24]. The company has since expanded its services into different cities and governorates in Jordan. Other OFD apps are generally less popular as they have a limited delivery coverage geographical area. The majority of OFD apps users in the present study were living in the central region of Jordan (Amman, Zarqa, Salt, Madaba), this, however, could be due to the availability of most delivery services in these four cities compared to other cities in Jordan.

In the present study, OFD apps users were more likely to be young adults with an age range of 18–25 years. Previous studies have indicated that young individuals use food delivery services more often [5, 8, 21, 25]. Older adults (over 55 years old) on the other hand are less likely to use OFD apps since they struggle to use smartphone devices and applications [26]. Moreover, they may have a higher tendency to use a more conventional mode of ordering food by contacting the restaurant directly.

The results of the present study are similar to those reported by Al Alwan (2020) in Jordan; where the majority of OFD apps users have a bachelor’s degree (46.3%) with a monthly income of less than 1000 JD (91.4%) [5]. More than half of the respondents in this study reported using OFD apps 1–3 times weekly. This is similar to a study among the Brazilian population, where more than a third of the respondents used OFD apps once or twice a week, and a similar proportion used OFD apps 1–3 times per month [13]. Another study indicated that the majority of university students used OFD apps more than once a week and 1–3 times monthly in China and Malaysia, respectively [22].

Most of the subjects in this study reported using OFD apps to order fast food more than any other available cuisines. This is not surprising as OFD apps usually make fast food outlets more accessible to users. For example, in China, Maimaiti et al. (2020) observed that 65% of the food outlets that provide food delivery services were fast food restaurants [27]. Similarly, Partridge et al. (2020) noticed that fast-food chains were the most popular food outlets and accounted for 38% of the studied OFD apps in Sydney and 54% in Auckland [28]. Available data indicates that general dietary patterns are in an energy surplus and shifting away from traditional foods with calorie-dense food options such as fast food and consuming soft drinks [29, 30]. Moreover, the low interest in ordering local food could be justified by the psychological attitude that affects consumers’ acceptance and consumption of certain food types. For example, some individuals show great pleasure in experiencing and eating new food [31]. Moreover, Gilliland reported that the majority (94%) of the respondent in their study desired to consume more local food, however, many of them are unable to obtain the foods they wanted either due to limited selection or difficulty finding them [32]. Eventually, these factors are bound to play a significant role in the expanding obesity rates and other NCDs [29, 30].

The findings of this study indicated that the preferred time to order meals among our participants was at lunch, which is typically the main meal in Jordan followed by dinner. The economic and business environment of Jordan has changed over the years and hectic lifestyles have made ordering food online a convenient option. According to Li et al. (2020), many Chinese workers do not have time to go out for lunch or prepare their meals [33], which is a similar trend in most countries globally. Moreover, the latter study suggested that a minimum of 48 minutes are saved upon each online food delivery order compared to time spent on grocery shopping, cooking, or residual cleaning [33]. This explains to a large extent the high dependency and usage of these applications among consumers.

The findings in the present study suggest that food-related attitudes and habits develop through interaction with others. Participants’ food choices in this study were influenced by their families, friends, and even by social media posts, magazines, TV, or YouTube advertisements. Tribhuvan (2020) studied factors influencing the use of OFD apps and observed that social media posts influenced 34% of the respondents while magazines, TV, or YouTube advertisements influenced 41% of the respondents. In the same study, nearly 44% of the respondents agreed that family and friends had influenced their food choices on OFD apps [23].

Social media marketing plays an important role in the area of food products. Social media and websites are major sources for obtaining information about the characteristics of individual food products [34]. Some marketing techniques used by OFD apps and restaurants are: photos, videos, links, prizes and giveaways, celebrities, sportspeople, vouchers, offers, and price promotions [35]. Advertisers on some social media platforms are now depending on interactive content; so, when consumers react to the advertised content a notification will appear on the consumers’ friends’ newsfeeds, timelines, and explorer, and by so spreading the advertising message to more people [36]. The majority of food marketing activities do not promote healthy food and are usually advocating mostly unhealthy foods [37]. Although all individuals are influenced by the power of marketing, adolescents and children are the most vulnerable groups because they may not successfully differentiate informative content from commercial content and are more sensitive to advertising materials [38, 39].

There is no doubt that OFD apps provide consumers with a diverse range of food options; however, many factors could contribute to an individual’s food choices. The cost of food is an important factor that influences food choices. Many food delivery services routinely offer discounts on food items or award free or reduced-price food delivery. This strategy pulls consumers who make buying decisions solely based on price. For such consumers, inexpensive foods are sometimes more pleasing despite the potential for diet-related illnesses [40]. The relatively low cost of unhealthy food [41] and the high cost of healthy food which approximates twice the price of unhealthier food per serving [42] were not considered as barriers for participants who look for healthy food choices in the present study. On the other hand, the appearance of the food sets expectations about taste, flavor, and healthiness of food which affect its acceptance and consumption [43]. Professional photographs of the menu served in restaurants on food delivery applications significantly appeal more to consumers, and can drastically increase consumers’ purchase intentions [44]. Also, it was reported that consumers build up criteria to judge the food based on the way it is plated and presented; aesthetic dishes were believed to be more natural and healthier [45, 46]. Despite this, participants of the present study seem to be aware that food styling can harm consumers by misleading healthiness judgments for unhealthy foods. Moreover, visuals can also include packaging elements including labels and materials. Wongprawmas et al. (2021) reported that consumers strongly believed that food should be packaged in an environmentally friendly manner, using minimal packaging materials [47]. Herrmann et al., (2022) highlighted that consumers are becoming increasingly aware of the impact of their food choices on the environment, are seeking out more sustainable options, and are willing to pay more for food that is packaged in environmentally friendly materials. This suggests that consumers may make food choices based on their beliefs about the environmental impact of the packaging, rather than on the quality of the food itself [48]. With regards to demographic characteristics of consumers (age, gender, educational level) and in contrast with the present study, Prakash and Pathak (2019) reported that younger generations of consumers exhibit a high sense of environmental consciousness and are actively concerned about the protection of the environment [49]. Moreover, Tüzemen and Kuru (2018) found that consumers with higher educational level have more environmental sensitivities [50]. On the other hand, another study showed that gender plays no role in the consumer perception of packaging [51].

Although food labels could be used as a tool to assist consumers in making healthy food choices, there is no such requirement in Jordan for restaurants and food delivery services. However, in Saudi Arabia, as a part of its vision of promoting healthy lifestyles, the local Food and Drug Authority obligated all food services establishments (digital and non-digital) to present food information (Calorie content) on their menus [52]. This strategy has shown promising results as Fakih et al. (2016) reported that the presence of food items including nutritional information, preparation, and ingredients was a factor driving consumers’ purchase intentions [53]. Christoph et al. found that looking for nutrition information was associated with healthy eating [54], these findings are in line with our results where food with displayed macronutrient content information was more likely to be considered when placing healthy food orders.

The delivery time is also a crucial factor that determines food choice while using OFD apps. The online food delivery environment requires highly efficient and scalable real-time delivery services [33]. In a study among OFD apps, the delay in delivery timings was reported as a concern when ordering food online by most customers [55]. Those who look for healthy food choices in the present study were not worried about the time of delivery. This could be justified by the widespread of fast food outlets which made it easier for consumers to choose the closer outlet to get meals delivered to their location in a relatively shortly. On the other hand, healthy food outlets are limited in number and often harder to find, therefore, participants who look for healthy food choices were aware that delivery times may be longer and vary depending on the location of the restaurant they ordered from.

The availability of healthy food options in OFD apps was another factor influencing food choice among the participants in the present study. Findings revealed that participants had difficulty finding nutritionally well-balanced dishes. Unfortunately, the digitalized food environment is often filled with unhealthy options. In Brazil, Horta et al. (2021) reported that restaurants on OFD apps provide consumers mainly with ultra-processed foods [56]. Moreover, amongst restaurants registered on OFD apps in Brazil, only 16% served vegetables (e.g. mix of salads and vegetable soup) [56]. In Australia and New Zealand, most food types available for delivery were considered unhealthy [28]. Furthermore, a study conducted in three international cities (Chicago, Amsterdam, and Melbourne) suggested that the most commonly advertised meals for home delivery are unhealthy foods; namely burgers and pizzas [57]. Consumption of food away from home is linked to poor diet quality (e.g. high levels of energy, total fat, sugar, and sodium) which lead to negative health impacts such as obesity and overweight [58]. A report by the World Health Organization (WHO) indicated that OFD apps increase the accessibility of poor-quality food and beverages (e.g. energy-dense, high fat, sugar, and sodium) [59]. Moreover, OFD apps are flagged as possibly contributing to the obesity problem, as they may encourage sedentary behavior [59]. A previously published study in China indicated that consumers’ weight gain, increased blood lipids, and gastrointestinal discomfort is caused by the long-term consumption of foods ordered from OFD apps [60].

The current study also observed that the abundant presence of vegetables in the food was associated with higher levels of perceived healthiness. A similar observation has been made previously among Americans revealing that 96.2% of respondents considered fresh vegetables to be healthy [61]. Furthermore, Jayne et al. (2018) reported that respondents who had greater vegetable intake possessed a healthy eating identity and consume an overall healthy diet [62]. For consumers who look for healthy food choices in our study, vegetables were not the only constituent of healthy food, however, healthy food choices were viewed comprehensively without solo focusing only on vegetables. The increasing usage of OFD apps has made both healthy and unhealthy food available at hand, with research considering those apps barriers to healthy food consumption. It is therefore imperative to highlight that linking consumer perception of healthy eating and investigating their related behavior is essential in enhancing dietary quality [63].

In the present study, participants’ attitudes toward food safety with regard to OFD apps were related to their level of concern about the safety of food packaging, food temperature, and delivery hygiene. In agreement to our findings, Wang et al. (2022) reported that a high hygiene rating of a particular restaurant increased ordering intentions among consumers [64]. Similarly, Vandenhaute et al., (2022) reported that consumers’ trust in food safety measures influences their willingness to purchase food products and are more likely to purchase food from establishments that they perceive to have high standards of hygiene and cleanliness [65]. A previously published study reported that the extent to which a consumer perceives a restaurant’s food as safe or unsafe depends on the relationship that has been established through prior experiences or the restaurant’s reputation [66]. In another study, it was observed that consumers were unwilling to return to a restaurant they deemed unsanitary [67]. The gender-based differences in perceptions of food safety and hygiene may stem from some differences in cultural norms, and/or personal experiences with foodborne illness. For instance, women in Jordan may be more likely to take on domestic responsibilities, including food preparation and safety, which could influence their perceptions of food safety and lead to more cautious attitudes towards food safety and hygiene. Females in Jordan tend to have more knowledge about food safety and are more likely to practice safe food handling behaviors [68].

Restaurants inspections in Jordan are conducted regularly by the Jordan Food and Drug Administration (JFDA), to ensure compliance with health requirements and safety measures [69]. In some countries such as the United Kingdom, there are some requirements for restaurants to join OFD apps; some of which are registered within the Food Standards Agency (FSA) and display the official FSA Food Hygiene Rating of the restaurant in the app [70]. While in Jordan, there are no such requirements, and OFD apps service providers had to maintain an appropriate hygiene level for safe food handling and delivery [69]. However, consumers in Jordan can still check the hygiene rate from other consumers’ generated comments, reviews, and feedback [65].

From a different perspective, Chandrasekhar et al. (2019) observed that not only did consumer perception of restaurant hygiene impact food choices, but also the hygienic status of food delivery [55]. For instance, meals that were delivered at sub-optimal temperatures were regarded as a reflection of poor delivery service, and not receiving a warm meal was associated with the food being stale. In the present study, it was observed that food delivered hot was regarded as freshly prepared and reflected higher quality and safety.

Moreover, most of our participants’ perception of the meal’s hygiene was affected by the appearance of the drivers. Similarly, Tran’s (2021) study which investigated the impact of food delivery hygiene among Vietnamese adults concluded that the hygiene of the driver positively affected the continued usage of the food delivery platform among consumers [71]. The appearance of food handlers has been identified as a key factor that affects consumers’ trust in the hygiene standards of food services [72].

Providing health education concerning dietary knowledge and practices may improve consumers’ knowledge and practices [73]. One way to favor the choice of healthy foods is through the community-based prevention marketing (CBPM) approach; which is a community-directed social change process that applies social marketing concepts and techniques to the development of health promotion and disease-prevention programs [73, 74]. The findings of the current study were subject to some limitations which are common in survey-based research. The use of an online self-administered questionnaire and the nature of the data collection method used may have led to a less representative sample. Also, the COVID-19 pandemic may have accelerated changes in consumer eating behavior with regard to the use of online orders of healthy choices as well as food safety and hygiene issues. However, while there is much uncertainty, transient changes in consumer behavior could persist for a long time. Finally, a limitation of using a convenience sample through the snowball method is the potential introduction of selection bias, which could restrict the broader applicability of the study’s findings. Despite the limitations, this study presents several strengths. This study is the first of its kind in the country tackling OFD apps usage and perceptions among consumers. Moreover, the findings of the present study identified gaps in healthy food knowledge among OFD apps users and provided room for future large-scale assessment and interventional studies aiming to increase awareness among consumers with regard to healthy and safe food.

## Conclusion

Online food delivery services are popular in Jordan; however, unhealthy eating patterns are widespread amongst OFD apps users. Considering the increasing prevalence rates of diet-related illnesses, the role of OFD apps could be concerning. Hence, initiatives should be established to spread awareness about nutritionally well-balanced diets and healthy eating. Healthy meals should be encouraged and well-advertised via these digital channels. Moreover, as food price, its appearance, packaging and labeling, and hygiene are key factors that determine consumer usage of OFD apps, local and global efforts should consider these factors when designing policies and implementing health awareness programs.

## Supporting information

S1 Table Participants attitudes towards food safety and delivery hygiene while ordering food through OFD apps (n = 675). (DOCX)

## References

[1] Ray A, Dhir A, Bala PK, Kaur P. Why do people use food delivery apps (FDA)? A uses and gratification theory perspective. Journal of Retailing and Consumer Services 2019;51:221–30. 10.1016/j.jretconser.2019.05.025.

[2] Granheim SI, Løvhaug AL, Terragni L, Torheim LE, Thurston M. Mapping the digital food environment: A systematic scoping review. Obesity Reviews 2022;23. doi: 10.1111/obr.13356 34519396

[3] Statista. Most popular Google Play app categories as of 1st quarter 2022 2022. https://www.statista.com/statistics/279286/google-play-android-app-categories/ (accessed July 22, 2022).

[4] Statista. Most popular Apple App Store categories as of 1st quarter 2022 2022. https://www.statista.com/statistics/270291/popular-categories-in-the-app-store/ (accessed July 22, 2022).

[5] Alalwan AA. Mobile food ordering apps: An empirical study of the factors affecting customer e-satisfaction and continued intention to reuse. International Journal of Information Management 2020;50:28–44. 10.1016/j.ijinfomgt.2019.04.008.

[6] Ahn J. Impact of cognitive aspects of food mobile application on customers’ behaviour. Current Issues in Tourism 2022;25:516–23. 10.1080/13683500.2021.1890700.

[7] Cha Seong-Soo, Seo Bo-Kyung. The Effect of Food Delivery Application on Customer Loyalty in Restaurant. Journal of Distribution Science 2020;18:5–12. 10.15722/JDS.18.4.202004.5.

[8] Stephens J, Miller H, Militello L. Food Delivery Apps and the Negative Health Impacts for Americans. Front Nutr 2020;7:14. doi: 10.3389/fnut.2020.00014 32154262 PMC7044187

[9] Deloitte. Delivering growth—The impact of third-party platform ordering on restaurants. 2019.

[10] Profeta A, Siddiqui SA, Smetana S, Hossaini SM, Heinz V, Kircher C. The impact of Corona pandemic on consumer’s food consumption: Vulnerability of households with children and income losses and change in sustainable consumption behavior. J Consum Prot Food Saf 2021;16:305–14. doi: 10.1007/s00003-021-01341-1 34421498 PMC8365131

[11] Sharma R, Dhir A, Talwar S, Kaur P. Over-ordering and food waste: The use of food delivery apps during a pandemic. International Journal of Hospitality Management 2021;96:102977. 10.1016/j.ijhm.2021.102977.

[12] Kumar S, Shah A. Revisiting food delivery apps during COVID-19 pandemic? Investigating the role of emotions. Journal of Retailing and Consumer Services 2021;62:102595. 10.1016/j.jretconser.2021.102595.

[13] Zanetta LD, Hakim MP, Gastaldi GB, Seabra LMJ, Rolim PM, Nascimento LGP, et al. The use of food delivery apps during the COVID-19 pandemic in Brazil: The role of solidarity, perceived risk, and regional aspects. Food Research International 2021;149:110671. doi: 10.1016/j.foodres.2021.110671 34600673 PMC8436220

[14] Tandon A, Kaur P, Bhatt Y, Mäntymäki M, Dhir A. Why do people purchase from food delivery apps? A consumer value perspective. Journal of Retailing and Consumer Services 2021;63:102667. 10.1016/j.jretconser.2021.102667.

[15] Goel T, Mujumdar V, Surya V, Kundlia Y, Saraf V, Bhardwaj S. Perception of Urban Consumers in Mumbai Towards Food Delivery Platforms. IJSRP 2020;10:109–20. 10.29322/IJSRP.10.07.2020.p10314.

[16] Almomani MH, Rababa M, Alzoubi F, Alnuaimi K, Alnatour A, Ali RA. Effects of a health education intervention on knowledge and attitudes towards chronic non‐communicable diseases among undergraduate students in Jordan. Nursing Open 2021;8:333–42. doi: 10.1002/nop2.634 33318841 PMC7729627

[17] PaquetteM-C. Perceptions of healthy eating: state of knowledge and research gaps. Canadian Journal of Public Health 2005;96. 16042159

[18] Bates S, Reeve B, Trevena H. A narrative review of online food delivery in Australia: challenges and opportunities for public health nutrition policy. Public Health Nutr 2020:1–11. doi: 10.1017/S1368980020000701 32515719 PMC7613985

[19] Lema K, Abuhay N, Kindie W, Dagne H, Guadu T. Food Hygiene Practice and Its Determinants Among Food Handlers at University of Gondar, Northwest Ethiopia, 2019. IJGM 2020;Volume 13:1129–37. doi: 10.2147/IJGM.S262767 33235486 PMC7679353

[20] World Health Organization. SLIDE TO ORDER: A FOOD SYSTEMS APPROACH TO MEAL DELIVERY APPs. 2021.

[21] Almansour FD, Allafi AR, Zafar TA, Al-Haifi AR. Consumer prevalence, attitude and dietary behavior of online food delivery applications users in Kuwait. Acta Biomedica Atenei Parmensis 2020;91:e2020178. doi: 10.23750/abm.v91i4.8543 33525272 PMC7927546

[22] Eu EZR, Sameeha MJ. Consumers’ Perceptions of Healthy Food Availability in Online Food Delivery Applications (OFD Apps) and Its Association With Food Choices Among Public University Students in Malaysia. Front Nutr 2021;8:674427. doi: 10.3389/fnut.2021.674427 34497818 PMC8419248

[23] Tribhuvan A. A STUDY ON CONSUMERS PERCEPTION ON FOOD APPS. International Journal of Advance Research and Innovative Ideas in Education 2020.

[24] Talabat. Our Story–Talabat Blog 2022. https://blog.talabat.com/our-story/ (accessed July 16, 2022).

[25] Lee SW, Sung HJ, Jeon HM. Determinants of Continuous Intention on Food Delivery Apps: Extending UTAUT2 with Information Quality. Sustainability 2019;11:3141. 10.3390/su11113141.

[26] Kim S, Yao W, Du X. Exploring Older Adults’ Adoption and Use of a Tablet Computer During COVID-19: Longitudinal Qualitative Study. JMIR Aging 2022;5:e32957. doi: 10.2196/32957 35134747 PMC8906838

[27] Maimaiti M, Ma X, Zhao X, Jia M, Li J, Yang M, et al. Multiplicity and complexity of food environment in China: full-scale field census of food outlets in a typical district. Eur J Clin Nutr 2020;74:397–408. doi: 10.1038/s41430-019-0462-5 31292528

[28] Partridge SR, Gibson AA, Roy R, Malloy JA, Raeside R, Jia SS, et al. Junk Food on Demand: A Cross-Sectional Analysis of the Nutritional Quality of Popular Online Food Delivery Outlets in Australia and New Zealand. Nutrients 2020;12:3107. doi: 10.3390/nu12103107 33053705 PMC7601596

[29] Musaiger AO. Overweight and Obesity in Eastern Mediterranean Region: Prevalence and Possible Causes. Journal of Obesity 2011;2011:1–17. doi: 10.1155/2011/407237 21941635 PMC3175401

[30] Ng SW, Zaghloul S, Ali H, Harrison G, Yeatts K, El Sadig M, et al. Nutrition transition in the United Arab Emirates. Eur J Clin Nutr 2011;65:1328–37. doi: 10.1038/ejcn.2011.135 21772317 PMC3304306

[31] Choe JY, Cho MS. Food neophobia and willingness to try non-traditional foods for Koreans. Food Quality and Preference 2011;22:671–7. 10.1016/j.foodqual.2011.05.002.

[32] Gilliland J, Sadler R, Clark A, O’Connor C, Milczarek M, Doherty S. Using a Smartphone Application to Promote Healthy Dietary Behaviours and Local Food Consumption. BioMed Research International 2015;2015:1–11. doi: 10.1155/2015/841368 26380298 PMC4561980

[33] Li C, Mirosa M, Bremer P. Review of Online Food Delivery Platforms and their Impacts on Sustainability. Sustainability 2020;12:5528. 10.3390/su12145528.

[34] Silva JM da, Rodrigues MB, Matos J de P, Mais LA, Martins APB, Claro RM, et al. Use of persuasive strategies in food advertising on television and on social media in Brazil. Preventive Medicine Reports 2021;24:101520. doi: 10.1016/j.pmedr.2021.101520 34976602 PMC8683935

[35] Freeman B, Kelly B, Baur L, Chapman K, Chapman S, Gill T, et al. Digital Junk: Food and Beverage Marketing on Facebook. Am J Public Health 2014;104:e56–64. doi: 10.2105/AJPH.2014.302167 25322294 PMC4232106

[36] Gaber HR, Wright LT. Fast-food advertising in social media. A case study on Facebook in Egypt. Journal of Business and Retail Management Research 2014;9:52–63.

[37] Meléndez-Illanes L, González-Díaz C, Álvarez-Dardet C. Advertising of foods and beverages in social media aimed at children: high exposure and low control. BMC Public Health 2022;22:1795. doi: 10.1186/s12889-022-14196-4 36138364 PMC9494888

[38] Szymański G. Marketing Activities of Local Food Producers in E-Commerce. Sustainability 2021;13:9406. 10.3390/su13169406.

[39] van der Bend DLM, Jakstas T, van Kleef E, Shrewsbury VA, Bucher T. Adolescents’ exposure to and evaluation of food promotions on social media: a multi-method approach. Int J Behav Nutr Phys Act 2022;19:74. doi: 10.1186/s12966-022-01310-3 35761362 PMC9235222

[40] Pilgrim-Hector J. Perception of Nutrition and Utilization of Healthy Food Ideas when Making Food Choices. 2016.

[41] Munt AE, Partridge SR, Allman-Farinelli M. The barriers and enablers of healthy eating among young adults: a missing piece of the obesity puzzle: A scoping review: Barriers and enablers of healthy eating. Obesity Reviews 2017;18:1–17. 10.1111/obr.12472. 27764897

[42] Kern D, Auchincloss A, Stehr M, Diez Roux A, Moore L, Kanter G, et al. Neighborhood Prices of Healthier and Unhealthier Foods and Associations with Diet Quality: Evidence from the Multi-Ethnic Study of Atherosclerosis. IJERPH 2017;14:1394. doi: 10.3390/ijerph14111394 29144387 PMC5708033

[43] Ueda J, Spence C, Okajima K. Effects of varying the standard deviation of the luminance on the appearance of food, flavour expectations, and taste/flavour perception. Sci Rep 2020;10:16175. doi: 10.1038/s41598-020-73189-8 32999406 PMC7528116

[44] Brewer P, Sebby AG. The effect of online restaurant menus on consumers’ purchase intentions during the COVID-19 pandemic. International Journal of Hospitality Management 2021;94:102777. doi: 10.1016/j.ijhm.2020.102777 34785837 PMC8588438

[45] Hagen L. Pretty Healthy Food: How and When Aesthetics Enhance Perceived Healthiness. Journal of Marketing 2021;85:129–45. 10.1177/0022242920944384.

[46] Zhang S, Qian J, Wu C, He D, Zhang W, Yan J, et al. Tasting More Than Just Food: Effect of Aesthetic Appeal of Plate Patterns on Food Perception. Foods 2022;11:931. doi: 10.3390/foods11070931 35407021 PMC8997541

[47] Wongprawmas R, Mora C, Pellegrini N, Guiné RPF, Carini E, Sogari G, et al. Food Choice Determinants and Perceptions of a Healthy Diet among Italian Consumers. Foods 2021;10:318. doi: 10.3390/foods10020318 33546323 PMC7913531

[48] Herrmann C, Rhein S, Sträter KF. Consumers’ sustainability-related perception of and willingness-to-pay for food packaging alternatives. Resources, Conservation and Recycling 2022;181:106219. 10.1016/j.resconrec.2022.106219.

[49] Prakash G, Pathak P. Intention to buy eco-friendly packaged products among young consumers of India: A study on developing nation. Journal of Cleaner Production 2017;141:385–93. 10.1016/j.jclepro.2016.09.116.

[50] Tüzemen A, Kuru Ö. Does the consumer want to be greened? The place of green packaging applications with green supply chain function in consumer perception. Journal of Cleaner Production 2018;8:200–16.

[51] Popovic I, Bossink BAG, Van Der Sijde PC. Factors Influencing Consumers’ Decision to Purchase Food in Environmentally Friendly Packaging: What Do We Know and Where Do We Go from Here? Sustainability 2019;11:7197. 10.3390/su11247197.

[52] Algheshairy RM, Alhomaid RM, Almujaydil MS, Alharbi HF, Alsanei WA. Influence of Using Food Delivery Applications on Adult Saudi Female Dietary Habits and Preferences during Covid-19 Lockdown Restrictions: Knowledge and Attitude Survey. In Review; 2022. 10.21203/rs.3.rs-1467008/v1. PMC9566569 36232068

[53] Fakih K, Assaker G, Assaf AG, Hallak R. Does restaurant menu information affect customer attitudes and behavioral intentions? A cross-segment empirical analysis using PLS-SEM. International Journal of Hospitality Management 2016;57:71–83. 10.1016/j.ijhm.2016.06.002.

[54] Christoph MJ, An R, Ellison B. Correlates of nutrition label use among college students and young adults: a review. Public Health Nutr 2016;19:2135–48. doi: 10.1017/S1368980015003183 26549218 PMC10271094

[55] Chandrasekhar N, Gupta S, Nanda N. Food Delivery Services and Customer Preference: A Comparative Analysis. Journal of Foodservice Business Research 2019;22:375–86. 10.1080/15378020.2019.1626208.

[56] Horta PM, Souza J de PM, Rocha LL, Mendes LL. Digital food environment of a Brazilian metropolis: food availability and marketing strategies used by delivery apps. Public Health Nutr 2021;24:544–8. doi: 10.1017/S1368980020003171 32900419 PMC10195591

[57] Poelman MP, Thornton L, Zenk SN. A cross-sectional comparison of meal delivery options in three international cities. Eur J Clin Nutr 2020;74:1465–73. doi: 10.1038/s41430-020-0630-7 32332863

[58] Halloran A, Faiz M, Chatterjee S, Clough I, Rippin H, Farrand C, et al. The cost of convenience: potential linkages between noncommunicable diseases and meal delivery apps. The Lancet Regional Health—Europe 2022;12:100293. doi: 10.1016/j.lanepe.2021.100293 35005677 PMC8717255

[59] WHO. WHO EUROPEAN REGIONAL OBESITY REPORT 2022. 2022.

[60] Dai X, Wu L, Hu W. Nutritional quality and consumer health perception of online delivery food in the context of China. BMC Public Health 2022;22:2132. doi: 10.1186/s12889-022-14593-9 36403009 PMC9675956

[61] Lusk JL. Consumer beliefs about healthy foods and diets. PLoS ONE 2019;14:e0223098. doi: 10.1371/journal.pone.0223098 31613889 PMC6793866

[62] Jayne JM, Frongillo EA, Torres-McGehee TM, Emerson DM, Glover SH, Blake CE. A Healthy Eating Identity is Associated with Healthier Food Choice Behaviors Among U.S. Army Soldiers. Military Medicine 2018;183:e666–70. doi: 10.1093/milmed/usy056 29635635

[63] Fernandez MA, Raine KD. Digital Food Retail: Public Health Opportunities. Nutrients 2021;13:3789. doi: 10.3390/nu13113789 34836044 PMC8624168

[64] Wang C, Li Y, Luo X, Fu H, Ye Z, Deng G. How Are Consumers Affected by Taste and Hygiene Ratings When Ordering Food Online? A Behavioral and Event-Related Potential Study. Front Neurosci 2022;16:844027. doi: 10.3389/fnins.2022.844027 35386593 PMC8978544

[65] Vandenhaute H, Gellynck X, De Steur H. COVID-19 Safety Measures in the Food Service Sector: Consumers’ Attitudes and Transparency Perceptions at Three Different Stages of the Pandemic. Foods 2022;11:810. doi: 10.3390/foods11060810 35327233 PMC8947567

[66] Isanovic S, Constantinides SV, Frongillo EA, Bhandari S, Samin S, Kenney E, et al. How Perspectives on Food Safety of Vendors and Consumers Translate into Food-Choice Behaviors in 6 African and Asian Countries. Current Developments in Nutrition 2023;7:100015. doi: 10.1016/j.cdnut.2022.100015 37181131 PMC10100931

[67] Worsfold D. Eating out: Consumer perceptions of food safety. International Journal of Environmental Health Research 2006;16:219–29. doi: 10.1080/09603120600641417 16611566

[68] Osaili TM, Al-Nabulsi AA, Taybeh AO. Food Safety Knowledge, Attitudes, and Practices Among Jordan Universities Students During the COVID-19 Pandemic. Front Public Health 2021;9:729816. doi: 10.3389/fpubh.2021.729816 34527655 PMC8435670

[69] JFDA. Home page 2022. http://www.jfda.jo/Default.aspx (accessed December 9, 2022).

[70] Taylor Wessing. The future of food delivery platforms 2021. https://www.taylorwessing.com/en/interface/2021/disruptive-tech-2021/the-future-of-food-delivery-platforms (accessed December 9, 2022).

[71] Tran VD. Using Mobile Food Delivery Applications during the COVID-19 Pandemic: Applying the Theory of Planned Behavior to Examine Continuance Behavior. Sustainability 2021;13:12066. 10.3390/su132112066.

[72] Bai L, Wang M, Yang Y, Gong S. Food safety in restaurants: The consumer perspective. International Journal of Hospitality Management 2019;77:139–46. 10.1016/j.ijhm.2018.06.023.

[73] Merritt RK, de Groot J, Almajali L, Patel N. Using Community-Based Prevention Marketing to Generate Demand for Healthy Diets in Jordan. Nutrients 2021;13:3068. doi: 10.3390/nu13093068 34578946 PMC8464799

[74] Bryant CA, Courtney AH, McDermott RJ, Lindenberger JH, Swanson MA, Mayer AB, et al. Community-Based Prevention Marketing for Policy Development: A New Planning Framework for Coalitions. Social Marketing Quarterly 2014;20:219–46. 10.1177/1524500414555948.

