# Correction: Healthy food and determinants of food choice on online food delivery applications

**DOI:** 10.1371/journal.pone.0296114

**Published:** 2023-12-14

**Authors:** Tareq M. Osaili, Anas A. Al-Nabulsi, Asma’ O. Taybeh, Leila Cheikh Ismail, Sheima T. Saleh

The order of the affiliations is incorrect. Please view the correct affiliation order here:

Tareq M. Osaili^1,2^, Anas A. Al-Nabulsi^2^, Asma’ O. Taybeh^2^, Leila Cheikh Ismail^1^, Sheima T. Saleh^1^

**1** Department of Clinical Nutrition and Dietetics, College of Health Sciences, University of Sharjah, P. O. Box 27272 Sharjah, United Arab Emirates, **2** Department of Nutrition and Food Technology, Faculty of Agriculture, Jordan University of Science and Technology, P.O. Box 3030, Irbid 22110, Jordan.
