# Supplementary material for: Healthy food and determinants of food choice on online food delivery applications
Source: PLoS One. 2023 Oct 19;18(10):e0293004. doi: 10.1371/journal.pone.0293004 (PMC10586623; doi:10.1371/journal.pone.0293004)
Supplement: S1 Table — (DOCX) [file pone.0293004.s001.docx]

**S1 Table**

| **Demographics** | | **Agree**  **N (%)** | **Neutral**  **N (%)** | **Disagree**  **N (%)** |
| --- | --- | --- | --- | --- |
| **“I believe that having the hygiene rating factor of the restaurant in the food application would be useful when ordering”** | | | | |
| **Gender** | |  |  |  |
|  | Female | 229 (33.9) | 205 (30.4) | 38 (5.6) |
|  | Male | 119 (17.6) | 69 (10.2) | 15 (2.2) |
| **Age** |  |  |  |  |
|  | 18-25 | 232 (34.4) | 179 (26.5) | 32 (4.7) |
|  | 26-35 | 66 (9.8) | 63 (9.3) | 16 (2.4) |
|  | 36-45 | 23 (3.4) | 19 (2.8) | 2 (0.3) |
|  | 46-55 | 14 (2.1) | 7 (1.0) | 3 (0.4) |
|  | >55 | 13 (1.9) | 6 (0.9) | 0 (0.0) |
| **Educational level** | |  |  |  |
|  | High school or less | 22 (3.3) | 19 (2.8) | 5 (0.7) |
|  | Diploma | 26 (3.9) | 11 (1.6) | 3 (0.4) |
|  | Bachelor’s degree | 269 (39.9) | 220 (32.6) | 39 (5.8) |
|  | Postgraduate | 31 (4.6) | 24 (3.6) | 6 (0.9) |
| **Residency** | |  |  |  |
|  | North Region | 71 (10.5) | 56 (8.3) | 11 (1.6) |
|  | Central Region | 244 (36.1) | 198 (29.3) | 39 (5.8) |
|  | South Region | 33 (4.9) | 20 (3.0) | 3 (0.4) |
| **Monthly allowance/income** | |  |  |  |
|  | <100 JD ^a^ | 93 (13.8) | 81 (12.0) | 17 (2.5) |
|  | 100-300 JD | 105 (15.6) | 84 (12.4) | 18 (2.7) |
|  | 301-500 JD | 68 (10.1) | 44 (6.5) | 9 (1.3) |
|  | 501-1000 JD | 52 (7.7) | 39 (5.8) | 5 (0.7) |
|  | >1000 JD | 30 (4.4) | 26 (3.9) | 4 (0.6) |
| **OFD apps usage frequency** | |  |  |  |
|  | Once a month | 121 (17.9) | 105 (15.6) | 20 (3.0) |
|  | Once a week | 99 (14.7) | 73 (10.8) | 16 (2.4) |
|  | 2-3 times/ week | 98 (14.5) | 60 (8.9) | 14 (2.1) |
|  | 4-6 times/ week | 16 (2.4) | 28 (4.1) | 2 (0.3) |
|  | Daily | 14 (2.1) | 8 (1.2) | 1 (0.1) |

| **“I believe that the items available are prepared and delivered under sanitary conditions”** | | | | |
| --- | --- | --- | --- | --- |
| **Gender** | |  |  |  |
|  | Female | 373 (55.3) | 66 (9.8) | 33 (4.9) |
|  | Male | 151 (22.4) | 40 (5.9) | 12 (1.8) |
| **Age** | |  |  |  |
|  | 18-25 | 335 (49.6) | 76 (17.2) | 32 (4.7) |
|  | 26-35 | 116 (17.2) | 18 (2.7) | 11 (1.6) |
|  | 36-45 | 37 (5.5) | 6 (0.9) | 1 (0.1) |
|  | 46-55 | 22 (3.3) | 1 (0.1) | 1 (0.1) |
|  | >55 | 14 (2.1) | 5 (0.7) | 0 (0.0) |
| **Educational level** | |  |  |  |
|  | High school or less | 35 (5.2) | 6 (0.9) | 5 (0.7) |
|  | Diploma | 34 (5.0) | 5 (0.7) | 1 (0.1) |
|  | Bachelor’s degree | 409 (60.6) | 86 (12.7) | 33 (4.9) |
|  | Postgraduate | 46 (6.8) | 9 (1.3) | 6 (0.9) |
| **Residency** | |  |  |  |
|  | North Region | 103 (15.3) | 26 (3.9) | 9 (1.3) |
|  | Central Region | 370 (54.8) | 78 (11.6) | 33 (4.9) |
|  | South Region | 51 (7.6) | 2 (0.3) | 3 (0.4) |
| **Monthly allowance/income** | |  |  |  |
|  | <100 JD ^a^ | 145 (21.5) | 30 (4.4) | 16 (2.4) |
|  | 100-300 JD | 161 (23.9) | 35 (5.2) | 11 (1.6) |
|  | 301-500 JD | 99 (14.7) | 14 (2.1) | 8 (1.2) |
|  | 501-1000 JD | 74 (11.0) | 18 (2.7) | 4 (0.6) |
|  | >1000 JD | 45 (6.7) | 9 (1.3) | 6 (0.9) |
| **OFD apps usage frequency** | |  |  |  |
|  | Once a month | 187 (27.7) | 44 (6.5) | 15 (2.2) |
|  | Once a week | 155 (23.0) | 25 (3.7) | 8 (1.2) |
|  | 2-3 times/ week | 130 (19.3) | 25 (3.7) | 17 (2.5) |
|  | 4-6 times/ week | 34 (5.0) | 8 (1.2) | 4 (0.6) |
|  | Daily | 18 (2.7) | 4 (0.6) | 1 (0.1) |

| **“I believe that the packaging of the meal influences my food choice”** | | | | |
| --- | --- | --- | --- | --- |
| **Gender** | |  |  |  |
|  | Female | 55 (8.1) | 114 (16.9) | 303 (44.9) |
|  | Male | 23 (3.4) | 53 (7.9) | 127 (18.8) |
| **Age** | |  |  |  |
|  | 18-25 | 282 (41.8) | 110 (16.3) | 51 (7.6) |
|  | 26-35 | 90 (13.3) | 33 (4.9) | 22 (3.3) |
|  | 36-45 | 29 (4.3) | 13 (1.9) | 2 (0.3) |
|  | 46-55 | 18 (2.7) | 4 (0.6) | 2 (0.3) |
|  | >55 | 11 (1.6) | 7 (1.0) | 1 (0.1) |
| **Educational level** | |  |  |  |
|  | High school or less | 31 (4.6) | 9 (1.3) | 6 (0.9) |
|  | Diploma | 30 (4.4) | 6 (0.9) | 4 (0.6) |
|  | Bachelor’s degree | 336 (49.8) | 136 (20.1) | 56 (8.3) |
|  | Postgraduate | 33 (4.9) | 16 (2.4) | 12 (1.8) |
| **Residency** | |  |  |  |
|  | North Region | 89 (13.2) | 38 (5.6) | 11 (1.6) |
|  | Central Region | 298 (44.1) | 121 (17.9) | 62 (9.2) |
|  | South Region | 43 (6.4) | 8 (1.2) | 5 (0.7) |
| **Monthly allowance/income** | |  |  |  |
|  | <100 JD ^a^ | 126 (18.7) | 42 (6.2) | 23 (3.4) |
|  | 100-300 JD | 133 (19.7) | 51 (7.6) | 23 (3.4) |
|  | 301-500 JD | 78 (11.6) | 27 (4.0) | 16 (2.4) |
|  | 501-1000 JD | 56 (8.3) | 33 (4.9) | 7 (1.0) |
|  | >1000 JD | 37 (5.5) | 14 (2.1) | 9 (1.3) |
| **OFD apps usage frequency** | |  |  |  |
|  | Once a month | 163 (24.1) | 63 (9.3) | 20 (3.0) |
|  | Once a week | 133 (19.7) | 38 (5.6) | 17 (2.5) |
|  | 2-3 times/ week | 97 (14.4) | 45 (6.7) | 30 (4.4) |
|  | 4-6 times/ week | 24 (3.6) | 15 (2.2) | 7 (1.0) |
|  | Daily | 13 (1.9) | 6 (0.9) | 4 (0.6) |

| **“I believe that having the meal delivered in environmentally friendly packaging materials influences my food choice”** | | | | |
| --- | --- | --- | --- | --- |
| **Gender** | |  |  |  |
|  | Female | 428 (63.4) | 35 (5.2) | 9 (1.3) |
|  | Male | 172 (25.5) | 26 (3.9) | 5 (0.7) |
| **Age** | |  |  |  |
|  | 18-25 | 390 (57.8) | 43 (6.4) | 10 (1.5) |
|  | 26-35 | 128 (19.0) | 13 (1.9) | 4 (0.6) |
|  | 36-45 | 40 (5.9) | 4 (0.6) | 0 (0.0) |
|  | 46-55 | 23 (3.4) | 1 (0.1) | 0 (0.0) |
|  | >55 | 19 (2.8) | 0 (0.0) | 0 (0.0) |
| **Educational level** | |  |  |  |
|  | High school or less | 40 (5.9) | 5 (0.7) | 1 (0.1) |
|  | Diploma | 35 (5.2) | 4 (0.6) | 1 (0.1) |
|  | Bachelor’s degree | 473 (70.1) | 46 (6.8) | 9 (1.3) |
|  | Postgraduate | 52 (7.7) | 6 (0.9) | 3 (0.4) |
| **Residency** | |  |  |  |
|  | North Region | 112 (16.6) | 22 (3.3) | 4 (0.6) |
|  | Central Region | 436 (64.6) | 36 (5.3) | 9 (1.3) |
|  | South Region | 52 (7.7) | 3 (0.4) | 1 (0.1) |
| **Monthly allowance/income** | |  |  |  |
|  | <100 JD ^a^ | 172 (25.5) | 14 (2.1) | 5 (0.7) |
|  | 100-300 JD | 178 (26.4) | 24 (3.6) | 5 (0.7) |
|  | 301-500 JD | 111 (16.4) | 8 (1.2) | 2 (0.3) |
|  | 501-1000 JD | 83 (12.3) | 11 (1.6) | 2 (0.3) |
|  | >1000 JD | 56 (8.3) | 4 (0.6) | 0 (0.0) |
| **OFD apps usage frequency** | |  |  |  |
|  | Once a month | 220 (32.6) | 22 (3.3) | 4 (0.6) |
|  | Once a week | 172 (25.5) | 12 (1.8) | 4 (0.6) |
|  | 2-3 times/ week | 147 (21.8) | 20 (3.0) | 5 (0.7) |
|  | 4-6 times/ week | 42 (6.2) | 4 (0.6) | 0 (0.0) |
|  | Daily | 19 (2.8) | 3 (0.4) | 1 (0.1) |

| **“The appearance of the driver (cleanliness, neatness) affects my perception of the meal’s hygiene**” | | | | |
| --- | --- | --- | --- | --- |
| **Gender** | |  |  |  |
|  | Female | 331 (49.0) | 91 (13.5) | 50 (7.4) |
|  | Male | 152 (22.5) | 31 (4.6) | 20 (3.0) |
| **Age** | |  |  |  |
|  | 18-25 | 310 (45.9) | 78 (11.6) | 55 (8.1) |
|  | 26-35 | 109 (16.1) | 26 (3.9) | 10 (1.5) |
|  | 36-45 | 30 (4.4) | 10 (1.5) | 4 (0.6) |
|  | 46-55 | 19 (2.8) | 4 (0.6) | 1 (0.1) |
|  | >55 | 15 (2.2) | 4 (0.6) | 0 (0.0) |
| **Educational level** | |  |  |  |
|  | High school or less | 32 (4.7) | 4 (0.6) | 10 (1.5) |
|  | Diploma | 29 (4.3) | 6 (0.9) | 5 (0.7) |
|  | Bachelor’s degree | 379 (56.1) | 100 (14.8) | 49 (7.3) |
|  | Postgraduate | 43 (6.4) | 12 (1.8) | 6 (0.9) |
| **Residency** | |  |  |  |
|  | North Region | 97 (14.4) | 29 (4.3) | 12 (1.8) |
|  | Central Region | 340 (50.4) | 85 (12.6) | 56 (8.3) |
|  | South Region | 46 (6.8) | 8 (1.2) | 2 (0.3) |
| **Monthly allowance/income** | |  |  |  |
|  | <100 JD ^a^ | 137 (20.3) | 34 (5.0) | 20 (3.0) |
|  | 100-300 JD | 142 (21.0) | 40 (5.9) | 25 (3.7) |
|  | 301-500 JD | 92 (13.6) | 15 (2.2) | 14 (2.1) |
|  | 501-1000 JD | 68 (10.1) | 21 (3.1) | 7 (1.0) |
|  | >1000 JD | 44 (6.5) | 12 (1.8) | 4 (0.6) |
| **OFD apps usage frequency** | |  |  |  |
|  | Once a month | 172 (25.5) | 51 (7.6) | 23 (3.4) |
|  | Once a week | 147 (21.8) | 28 (4.1) | 13 (1.9) |
|  | 2-3 times/ week | 123 (18.2) | 26 (3.9) | 23 (3.4) |
|  | 4-6 times/ week | 27 (4.0) | 11 (1.6) | 8 (1.2) |
|  | Daily | 14 (2.1) | 6 (0.9) | 3 (0.4) |

| **“The temperature of the meal when delivered mainly gives me an impression about the quality of the food** “ | | | | |
| --- | --- | --- | --- | --- |
| **Gender** | |  |  |  |
|  | Female | 421 (62.4) | 24 (3.5) | 27 (4.0) |
|  | Male | 184 (27.3) | 11 (1.6) | 8 (1.2) |
| **Age** | |  |  |  |
|  | 18-25 | 385 (57.0) | 28 (4.1) | 30 (4.4) |
|  | 26-35 | 136 (20.1) | 5 (0.7) | 4 (0.6) |
|  | 36-45 | 42 (6.2) | 2 (0.3) | 0 (0.0) |
|  | 46-55 | 24 (3.6) | 0 (0.0) | 0 (0.0) |
|  | >55 | 18 (2.7) | 0 (0.0) | 1 (0.1) |
| **Educational level** | |  |  |  |
|  | High school or less | 42 (6.2) | 2 (0.3) | 2 (0.3) |
|  | Diploma | 35 (5.2) | 2 (0.3) | 3 (0.4) |
|  | Bachelor’s degree | 469 (69.5) | 30 (4.4) | 29 (4.3) |
|  | Postgraduate | 59 (8.7) | 1 (0.1) | 1 (0.1) |
| **Residency** | |  |  |  |
|  | North Region | 116 (17.2) | 12 (1.8) | 10 (1.5) |
|  | Central Region | 436 (64.6) | 22 (3.3) | 23 (3.4) |
|  | South Region | 53 (7.9) | 1 (0.1) | 2 (0.3) |
| **Monthly allowance/income** | |  |  |  |
|  | <100 JD ^a^ | 169 (25.0) | 10 (1.5) | 12 (1.8) |
|  | 100-300 JD | 175 (25.9) | 18 (2.7) | 14 (2.1) |
|  | 301-500 JD | 113 (16.7) | 3 (0.4) | 5 (0.7) |
|  | 501-1000 JD | 90 (13.3) | 3 (0.4) | 3 (0.4) |
|  | >1000 JD | 58 (8.6) | 1 (0.1) | 1 (0.1) |
| **OFD apps usage frequency** | |  |  |  |
|  | Once a month | 220 (32.6) | 11 (1.6) | 15 (2.2) |
|  | Once a week | 176 (26.1) | 6 (0.9) | 6 (0.9) |
|  | 2-3 times/ week | 151 (22.4) | 11 (1.6) | 10 (1.5) |
|  | 4-6 times/ week | 38 (5.6) | 6 (0.9) | 2 (0.3) |
|  | Daily | 20 (3.0) | 1 (0.1) | 2 (0.3) |

| **“The temperature of the meal when delivered mainly gives me an impression about the safety of the meal** “ | | | | |
| --- | --- | --- | --- | --- |
| **Gender** | |  |  |  |
|  | Female | 372 (55.1) | 60 (8.9) | 40 (5.9) |
|  | Male | 173 (25.6) | 19 (2.8) | 11 (1.6) |
| **Age** | |  |  |  |
|  | 18-25 | 337 (49.9) | 62 (9.2) | 44 (6.5) |
|  | 26-35 | 128 (19.0) | 12 (1.8) | 5 (0.7) |
|  | 36-45 | 40 (5.9) | 4 (0.6) | 0 (0.0) |
|  | 46-55 | 23 (3.4) | 1 (0.1) | 0 (0.0) |
|  | >55 | 17 (2.5) | 0 (0.0) | 2 (0.3) |
| **Educational level** | |  |  |  |
|  | High school or less | 34 (5.0) | 9 (1.3) | 3 (0.4) |
|  | Diploma | 35 (5.2) | 2 (0.3) | 3 (0.4) |
|  | Bachelor’s degree | 424 (62.8) | 63 (9.3) | 41 (6.1) |
|  | Postgraduate | 52 (7.7) | 5 (0.7) | 4 (0.6) |
| **Residency** | |  |  |  |
|  | North Region | 102 (15.1) | 23 (3.4) | 13 (1.9) |
|  | Central Region | 392 (58.1) | 53 (7.9) | 36 (5.3) |
|  | South Region | 51 (7.6) | 3 (0.4) | 2 (0.3) |
| **Monthly allowance/income** | |  |  |  |
|  | <100 JD ^a^ | 156 (23.1) | 23 (3.4) | 12 (1.8) |
|  | 100-300 JD | 159 (23.6) | 29 (4.3) | 19 (2.8) |
|  | 301-500 JD | 98 (14.5) | 12 (1.8) | 11 (1.6) |
|  | 501-1000 JD | 79 (11.7) | 11 (1.6) | 6 (0.9) |
|  | >1000 JD | 53 (7.9) | 4 (0.6) | 3 (0.4) |
| **OFD apps usage frequency** | |  |  |  |
|  | Once a month | 205 (30.4) | 25 (3.7) | 16 (2.4) |
|  | Once a week | 156 (23.1) | 21 (3.1) | 11 (1.6) |
|  | 2-3 times/ week | 138 (20.4) | 17 (2.5) | 17 (2.5) |
|  | 4-6 times/ week | 30 (4.4) | 11 (1.6) | 5 (0.7) |
|  | Daily | 16 (2.4) | 5 (0.7) | 2 (0.3) |
